# Treatment Outcomes for Patients with Extensively Drug-Resistant Tuberculosis, KwaZulu-Natal and Eastern Cape Provinces, South Africa

**DOI:** 10.3201/eid2209.160084

**Published:** 2016-09

**Authors:** Charlotte L. Kvasnovsky, J. Peter Cegielski, Martie L. van der Walt

**Affiliations:** University of Maryland Medical Center, Baltimore, Maryland, USA (C.L. Kvasnovsky);; Medical Research Council of South Africa, South Africa (C.L. Kvasnovsky, M.L. van der Walt);; Centers for Disease Control and Prevention, Atlanta, Georgia, USA (J.P. Cegielski)

**Keywords:** tuberculosis and other mycobacteria, bacteria, extensively drug-resistant tuberculosis, XDR TB, HIV/AIDS, epidemiology, treatment outcomes, antimicrobial resistance, KwaZulu-Natal Province, Eastern Cape Province, South Africa

## Abstract

We analyzed data for a retrospective cohort of patients treated for extensively drug-resistant tuberculosis in 2 provinces in South Africa and compared predictors of treatment outcome in HIV-positive patients who received or had not received antiretroviral drugs with those for HIV-negative patients. Overall, 220 (62.0%) of 355 patients were HIV positive. After 2 years, 34 (10.3%) of 330 patients with a known HIV status and known outcome had a favorable outcome. Multivariate analysis showed that predictors of favorable outcome were negative results for acid-fast bacilli by sputum microscopy at start of treatment and weight >50 kg. HIV-positive patients were more likely to have an unfavorable outcome. The strongest predictor of unfavorable outcome was weight <50 kg. Overall outcomes were poor. HIV status was not a predictor of favorable outcome, but HIV-positive patients were more likely to have an unfavorable outcome. These results underscore the need for timely and adequate treatment for tuberculosis and HIV infection.

## Abstract 2

Results underscore the need for timely and adequate treatment for tuberculosis and HIV/AIDS.

Tuberculosis (TB) remains a major cause of illness and death in the 21st century. There were an estimated 9.6 million incident cases worldwide in 2014 (*1*). In addition, an estimated 3.3% of new cases and 20% of retreatment cases are multidrug-resistant TB (MDR TB), which is defined as TB resistant to at least rifampin and isoniazid, the 2 most powerful first-line drugs. This resistance threatens global TB control efforts. MDR TB patients need access to treatment, require longer treatment with toxic medications, and have a lower probability of cure.

Globally, MDR TB has a treatment success rate of 50% (*1*). Extensively drug-resistant TB (XDR TB) is MDR TB with additional resistance to at least a fluoroquinolone and any 1 of 3 injectable second-line drugs (amikacin, capreomycin, or kanamycin). By 2014, XDR TB had been reported in 105 countries, including 10 countries in sub-Saharan Africa (*1*).

Shortly after XDR TB was first described in 2005, patients in 1 hospital in KwaZulu-Natal Province were shown to have extremely rapid and high mortality rates in a setting with high HIV prevalence (*2*). Since that time, published case series have shown higher cure rates (48%–60%), and the World Health Organization (WHO) reported a treatment success rate of 22% for reported XDR TB cases worldwide (*3**,**4*). However, these studies included few HIV-positive patients.

We previously reported poor outcomes for patients with XDR TB after 1 year of treatment (*5*). Thus, we sought to collect the largest possible dataset for patients with XDR TB in an area of high HIV prevalence to assess predictors of favorable and unfavorable treatment outcomes among patients.

## Patients and Methods

We conducted a retrospective cohort study of patients initiating treatment for XDR TB in 2 contiguous provinces in South Africa. In Eastern Cape Province, all patients given a diagnosis of XDR TB by the provincial public laboratory were reported to the XDR TB treatment facility as described (*6*). Therefore, our sample in this province consisted of continuous patients who were given a diagnosis of XDR TB during October 1, 2006–January 31, 2008. We also included continuous patients initiating treatment for XDR TB in this province during April 1–July 1, 2008, who were eligible for treatment with moxifloxacin after this drug became available in this province. In KwaZulu-Natal Province, new XDR TB case-patients were reported to individual clinics, which then contacted the sole XDR TB treatment facility to place their patients on a list to initiate treatment. Our sample in KwaZulu-Natal Province included all patients who initiated XDR TB treatment during October 1, 2006–January 31, 2008. Ethical approval to conduct this research was obtained from the Medical Research Council of South Africa, Eastern Cape Department of Health, and KwaZulu-Natal Department of Health.

In accordance with national and provincial policy during the study, patients with XDR TB initiated treatment only as inpatients at specialized referral hospitals. Inpatient XDR TB treatment was offered free of charge; however, this treatment was dependent on bed availability and often resulted in delays before treatment initiation (*7*). At provider discretion, generally upon completion of the intensive phase of treatment and after >2 consecutive negative sputum samples, patients were discharged from the hospital. After discharge, ambulatory patients were followed up at local clinics in Eastern Cape Province and at a hospital-based clinic in KwaZulu-Natal Province.

### Case Definition

Patients were given a diagnosis of XDR TB if any sputum sample showed resistance to rifampin, isoniazid, a fluoroquinolone, and any injectable second-line drug. Drug-susceptibility testing was performed by local National Health Laboratory Services facilities for 1 or several samples. All patients had >1 subsequent positive sputum culture. In Eastern Cape Province, samples were tested for resistance to rifampin, isoniazid, ethambutol, ethionamide, streptomycin, amikacin, ofloxacin, and, starting in early 2008, capreomycin. In KwaZulu-Natal Province, samples were tested for resistance to rifampin, isoniazid, ethambutol, streptomycin, kanamycin, and ciprofloxacin.

Voluntary counseling and testing for HIV was offered to all patients. In accordance with national policy during the study, HIV-positive, drug-resistant TB patients who were not receiving antiretroviral drugs (ARVs) were given these drugs either at completion of the 4-month intensive phase of drug-resistant TB treatment if they had a CD4 lymphocyte count <200 cells/mm^3^ or as soon as their CD4 lymphocyte count was <200 cells/mm^3^. ARV treatment included 3 drugs in accordance with South African National Guidelines. Patients who initiated ARV therapy >30 days after XDR TB treatment initiation were analyzed as patients not receiving ARVs at the time of starting XDR TB treatment.

### Outcome Definitions

We applied standard MDR TB case definitions for XDR TB: favorable treatment outcomes were cure and treatment completion, and unfavorable treatment outcomes were death, loss to follow-up, and treatment failure (*4**,**8*). We applied these outcomes on the basis of the status of the patient 2 years (730 days) after treatment initiation. Patients who had a poor prognosis and were subsequently lost to follow-up were included among poor outcomes.

Patients who had 2 consecutive negative sputum cultures obtained >30 days apart after treatment initiation were considered to have achieved culture conversion. Initial time to sputum culture conversion was calculated as the interval in days between the date of treatment initiation for XDR TB and the collection date for the first of 2 consecutive negative sputum cultures.

### Treatment of XDR TB

Patients were given individualized regimens composed of first-line and second-line TB drugs available within their province: ethambutol, pyrazinamide, high-dose isoniazid, capreomycin, para-amino salicylic acid, moxifloxacin, cycloserine, and terizidone. Cycloserine and terizidone were considered interchangeable. These drugs were supplemented by WHO Group 5 drugs (amoxicillin/clavulanic acid and clarithromycin) on an individual basis.

Patients were considered to be receiving effective treatment if they received >4 drugs to which their TB could be considered susceptible per WHO guidelines (*9*). A drug was considered effective if 1) it was recognized as an agent for treatment of TB; 2) the patient had either never received it or received it for <3 months before XDR TB treatment; and 3) patient isolates were not found to be resistant to the drug by drug-susceptibility testing.

While hospitalized, patients received directly observed therapy (DOT), although the quality of hospital-delivered DOT was unknown. Once discharged, many patients continued to receive DOT through various delivery models, and others were seen monthly for medication refill and to give sputum samples for smear and culture testing.

### Data Collection and Analysis

Clinical and treatment data were abstracted from patient medical records at XDR TB treatment hospitals from multiple-site visits; all data were censored on March 10, 2010. Hospital-based HIV treatment registers were also reviewed for any additional follow-up information.

Double-data entry was performed on a database created for this study in Epi Info version 3.3.2 (Centers for Disease Control and Prevention, Atlanta, GA, USA). Data cleaning was performed by using EpiInfo and Stata 10 (StataCorp LP, College Station, TX, USA). Analysis was performed by using SAS 9.1 and SAS 9.4 (SAS Institute, Inc., Cary, NC, USA).

Univariate and bivariate analyses were performed to identify differences between HIV-negative patients, HIV-positive patients receiving ARVs, HIV-positive patients not receiving ARVs at initiation of treatment, and HIV-positive patients with unknown status for ARVs. Categorical data were compared by using χ^2^ or Fisher exact tests as appropriate. Continuous variables were compared across these 3 categories by using PROC GLM (SAS Institute, Inc.) and the F statistic.

For comparison of unadjusted deaths, we used a Kaplan–Meier plot and log-rank test that were stratified by HIV infection and ARV treatment status and excluded patients with unknown status for ARVs. We used death as the event variable and censored all other outcomes at 730 days.

We sought to further assess risk factors for favorable and unfavorable treatment outcomes. At treatment day 90, survival curves for HIV-positive patients receiving ARVs and those not receiving ARVs crossed, which visually violated the proportionality assumption necessary for Cox proportional hazards. We confirmed this finding by plotting Schoenfeld residuals over time, for which there was a relationship, and by creating a time-varying covariate among only HIV-positive patients, for which the interaction term was a significant predictor in the model (*10**,**11*).

However, when we compared only HIV-positive patients with and without ARV treatment, we found no difference in outcome, and models comparing these 2 groups did not predict treatment outcome. Thus, we combined HIV-positive patients with and without ARV treatment in the final model. In this instance, the proportionality assumption was met.

Because of covariance between effective treatment and previous MDR TB treatment >6 months, we included only previous MDR TB treatment in the multivariate model. All tests were 2-sided, and p<0.05 was considered statistically significant.

## Results

### Baseline Characteristics

A total of 355 patients were included in the analysis: 229 (64.5%) from Eastern Cape Province and 126 (35.5%) from KwaZulu-Natal Province (Table 1). Most (194, 54.6%) patients were women, and median age was 35 years (interquartile range [IQR] 28–44 years) at start of treatment.

**Table 1:** Baseline characteristics of patients initiating treatment for extensively drug-resistant tuberculosis, KwaZulu-Natal and Eastern Cape Provinces, South Africa, 2006–2010*

Characteristic	Total, n = 355	HIV negative, n = 124	HIV positive, receiving ARVs at start of treatment, n = 114	HIV positive, not receiving ARVs at start of treatment, n = 79	HIV positive, ARV status unknown, n = 27	p value†
Treated in KZN	124 (36.1)	28 (22.6)	57 (46.0)	34 (27.4)	5 (4.0)	**<0.0001**
Treated in EC	220 (64.0)	96 (43.6)	57 (25.9)	45 (20.5)	22 (10.0)	
Male sex†	155 (45.1)	74 (47.7)	41 (26.5)	29 (18.7)	11 (7.1)	**0.0007**
Age, y, at start of treatment	35 (28–44)	37 (24–48)	35 (30–41)	34 (30–42)	34 (28–42)	0.44
Weight, kg, at start of treatment	49 (43–55)	49 (43–55)	49 (44–57)	49 (43–54)	46 (42–53)	0.52
Weight >50 kg at start of treatment	176 (51.2)	65 (36.9)	59 (33.5)	37 (21.0)	15 (8.5)	0.83
Diabetes	14 (4.1)	10 (71.4)	1 (7.1)	3 (21.4)	0	**0.03**
Initial CD4 count, cells/mm^3^	220 (64.0)	0	114 (51.8)	79 (35.9)	27 (12.3)	**<0.0001**
AIDS at start of treatment‡	193 (110–313)	NA	181 (97–238)	217 (126–370)	329 (162–413)	**0.002**
Months previous MDR TB treatment	4 (0–8)	6 (0–11)	2 (0–7)	1 (0–7)	5.5 (4–16)	0.62
Months previous TB treatment	13 (8–19)	13 (8–21)	12 (8–17)	12 (7–18)	16 (11–24)	0.44
Previous TB episodes	2 (2–3)	3 (2–3)	2 (2–3)	2 (2–3)	3 (2–4)	0.34
Previous episode of MDR TB	214 (63.3)	84 (39.3)	64 (29.9)	43 (20.1)	23 (10.8)	**0.004**
Cavitary disease at start of treatment	187 (57.5)	81 (43.3)	55 (29.4)	37 (19.8)	14 (7.5)	**0.05**
Smear positive at start of treatment	173 (53.1)	48 (27.8)	67 (38.7)	41 (23.7)	17 (9.8)	**0.03**
No. TB-resistant drugs	5 (4–6)	4 (4–5)	5 (4–6)	5 (4–6)	5 (5–6)	**0.005**

*Values are no. (%) or median (interquartile range). p values in bold are statistically significant. ARVs, antiretroviral drugs; EC, Eastern Cape Province; KZN, KwaZulu Natal Province; MDR TB, multidrug-resistant tuberculosis; NA, not applicable; TB, tuberculosis. †Calculation excluded 11 patients with unknown HIV status. ‡AIDS defined by CD4 count <200 cells/mm^3^ at start of treatment or AIDS-defining illness other than TB.

Eleven patients did not have a known HIV status, and 220 (62.0%) patients were HIV positive at start of treatment. Of these patients, 114 (51.8%) were receiving ARVs at start of TB treatment, and 79 (35.9%) were not. Of the 79 patients not receiving ARVs at start of TB treatment, 23 (10.5%) initiated ARVs during XDR TB treatment, after a median of 224 days (IQR 89–507 days). An additional 27 (12.3%) patients had an unknown ARV status.

Most (97.7%) patients had >1 month of TB treatment before initiating XDR TB treatment. HIV-negative patients were similar to HIV-positive patients in this respect; they had ≈1 year of previous TB treatment before initiating XDR TB treatment. Patients had a median of 13 previous months (IQR 8–19 months) of TB treatment, including a median of 8 months (IQR 5–12 months) of category I TB treatment (2 months of rifampicin, isoniazid, ethambutol, and pyrazinamide [intensive phase], followed by 4 months of rifampicin and isonazid alone [continuous phase], with extension per treating clinician) (*12*) for presumed drug-susceptible TB and 4 months (IQR 0–8 months) of MDR TB treatment. We found no difference in duration of category I treatment between groups (p = 0.86), but HIV-negative patients were more likely than HIV-positive patients to have had a previous episode of MDR TB (p = 0.004).

HIV-negative patients were least likely to have smear positive disease (42.1%) when compared with HIV-positive patients receiving ARVs (60.9%), HIV-positive patients not receiving ARVs at start of treatment (54.7%), and HIV-positive patients with an unknown ARV status (63.0%; p = 0.02). Cavitary disease was more frequent in HIV-negative patients (67.5%) than in HIV-positive patients receiving ARVs (50.9%), HIV-positive patients not receiving ARVs at start of treatment (51.4%), and HIV-positive patients with unknown ARV status (56.0%; p = 0.05).

### Treatment Outcomes

After 2 years of treatment, 330 (95.9%) patients with a known HIV status had a known treatment outcome. Of these patients, 21 (6.4%) met the definition for cure and 13 (3.9%) met the definition for treatment completion; therefore, a total of 34 (10.3%) patients had a favorable treatment outcome (Table 2). An additional 61 (18.5%) patients were alive but showed treatment failure after 2 years. A total of 211 (63.9%) patients died, and 24 (7.3%) patients interrupted their treatment prematurely and were lost to follow-up.

**Table 2:** Treatment and treatment outcomes for patients with extensively drug-resistant tuberculosis, KwaZulu-Natal and Eastern Cape Provinces, South Africa, 2006–2010*

Characteristic	Total, n = 355	HIV negative, n = 124	HIV positive, receiving ARVs, n = 114	HIV positive, not receiving ARVs, n = 79	HIV positive, ARV status unknown, n = 27	p value†
No. drugs in treatment regimen	5 (5–6)	5 (5)	5 (5–6)	5 (5)	5 (5–6)	0.56
Effective drug treatment	109 (31.7)	29 (26.6)	46 (42.2)	32 (29.4)	2 (1.8)	**0.0004**
Any culture conversion	77 (22.4)	28 (22.6)	33 (29.0)	12 (15.2)	4 (14.8)	0.11
Alive after 2 y treatment	96 (27.9)	46 (37.1)	30 (26.3)	18 (22.8)	2 (7.4)	**0.007**
Favorable treatment outcome	34 (10.3)	15 (12.3)	13 (12.2)	5 (6.6)	1 (4.0)	0.37

*Values are median (interquartile range) or no. (%). p values in bold are statistically significant. ARVs, antiretroviral drugs. †Calculation excluded 11 patients with unknown HIV status.

**Figure 1:**
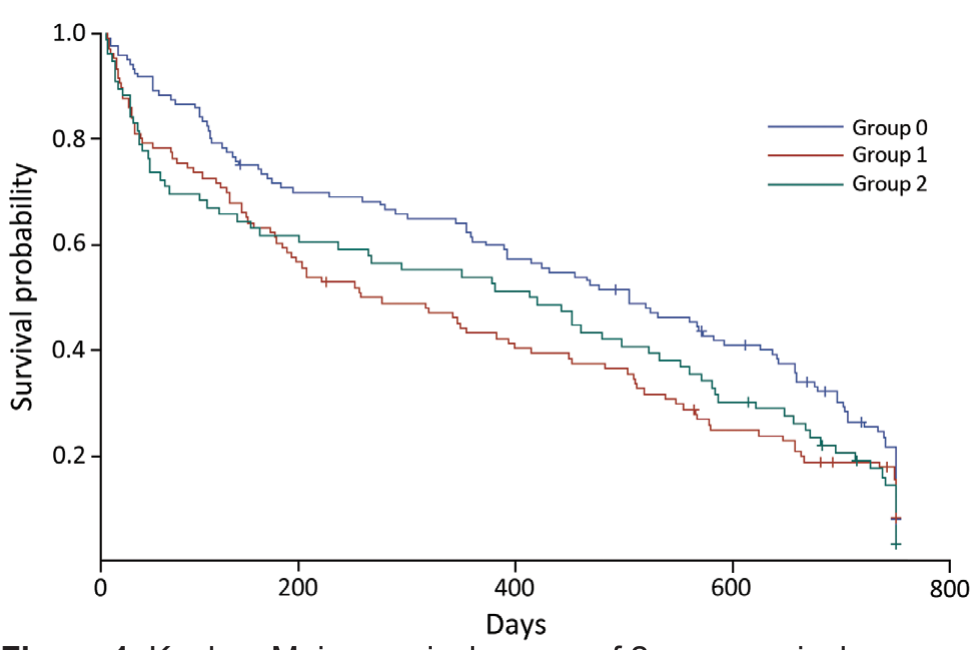
Kaplan–Meier survival curves of 2-year survival probability (product limit survival estimates) for patients with extensively drug-resistant tuberculosis, KwaZulu-Natal and Eastern Cape Provinces, South Africa, 2006–2010. Group 0, HIV-negative patients; group 1, HIV-positive patients receiving antiretroviral drugs at start of treatment; group 2: HIV-positive patients not receiving antiretroviral drugs at start of treatment. +, censored value.

We found no significant difference in 2-year survival by Kaplan-Meier survival curves between the 3 groups (p = 0.07, by log-rank test) (Figure 1). There was also no significant difference in 2-year survival between HIV-positive patients with or without ARV treatment (p = 0.89). In pairwise comparisons, we found that the only difference between the 3 groups was that HIV-positive patients receiving ARVs at start of treatment had better survival rates than HIV-positive patients not receiving ARVs (p = 0.04).

We compared patients who initiated treatment early in the cohort, immediately after XDR TB was first reported and explicitly treated (October 2006–February 2007), with patients treated in the last 6 months of the cohort. We found no improvement in sputum culture conversion (p = 0.46) or favorable outcome (p = 0.70).

HIV-positive patients who initiated ARV treatment during XDR TB treatment had a median baseline CD4 count of 192 cells/mm^3^ (IQR 118–236 cells/mm^3^). We found no difference in favorable treatment outcomes among HIV-positive patients who initiated ARV treatment before or after start of XDR TB treatment (p = 0.59).

### Culture Conversion

Of 78 patients who achieved culture conversion during the study, 71 had a known treatment outcome. Of these, 38 (53.5%) had culture conversion within 4 months of start of treatment, and an additional 20 (81.7%) patients had culture conversion within 8 months of start of treatment.

**Figure 2:**
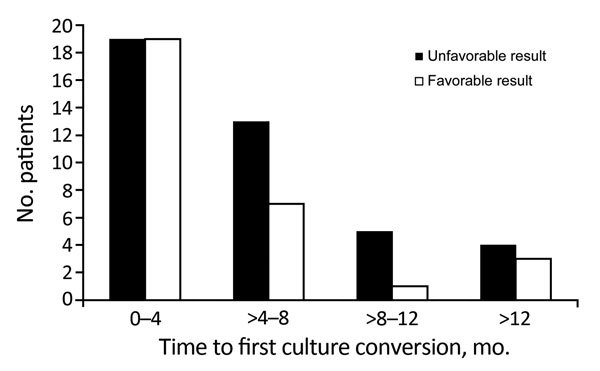
Favorable and unfavorable treatment outcomes for patients with extensively drug-resistant tuberculosis, according to time-to-first sputum culture conversion, KwaZulu-Natal and Eastern Cape Provinces, South Africa, 2006–2010.

However, even patients who achieved sputum culture conversion had poor treatment outcomes overall: 39 (55.7%) patients had unfavorable treatment outcomes (11 patients died, 10 were lost to follow-up, and 18 were alive but showed treatment failure with persistently positive sputum cultures). We found no difference in favorable treatment outcome between patients with early culture conversion (within either 4 months or 8 months of start of treatment) and patients with culture conversion after 8 months (p = 0.16 and p = 0.35, respectively) (Figure 2).

### Multivariate Predictors of Favorable Treatment Outcome

Multivariate analysis showed that HIV status was not predictive of a favorable outcome (Table 3). Smear-negative patients were nearly 3-fold more likely to have a favorable treatment outcome (hazard ratio [HR] 2.69, 95% CI 1.29–5.63, p = 0.009). Patients who weighed >50 kg at start of treatment were nearly 4-fold more likely to have a favorable outcome (HR 3.64, 95% CI 1.68–7.92, p = 0.001).

**Table 3:** Predictors of favorable outcome among patients initiating treatment for extensively drug-resistant tuberculosis, KwaZulu-Natal and Eastern Cape Provinces, South Africa, 2006–2010*

Predictor	Unadjusted analysis			Multivariate analysis	
	HR (95% CI)	p value		HR (95% CI)	p value
HIV positive	1.08 (0.59–1.98)	0.81		1.29 (0.60–2.75)	0.52
Previous MDR TB treatment	0.59 (0.33–1.06)	0.08		0.77 (0.35–1.66)	0.50
Smear negative	2.97 (1.41–6.26)	**0.004**		2.69 (1.29–5.63)	**0.009**
Age	0.99 (0.97–1.02)	0.55		0.99 (0.96–1.02)	0.58
Weight >50 kg	1.93 (0.93–4.02)	0.08		3.64 (1.68–7.92)	**0.001**
KwaZulu Province	1.98 (1.12–3.52)	0.02		1.69 (0.81–3.53)	0.16
Male sex	0.57 (0.30–1.09)	0.09		0.77 (0.36–1.67)	0.51
Cavitary disease by chest radiograph	0.61 (0.33–1.10)	0.10		0.50 (0.24–1.03)	0.06

*p values in bold are statistically significant. HR, hazard ratio; MDR TB, multidrug-resistant tuberculosis.

### Multivariate Predictors of Unfavorable Treatment Outcome

HIV-positive patients were more likely to have an unfavorable treatment outcome (adjusted HR 1.35, 95% CI 1.06–1.72, p = 0.02) (Table 4). Weight <50 kg at start of treatment was the strongest predictor of an unfavorable treatment outcome for all patients (adjusted HR 1.56, 95% CI 1.23–1.98, p = 0.0003).

**Table 4:** Predictors of unfavorable outcome among patients initiating treatment for extensively drug-resistant tuberculosis, KwaZulu-Natal and Eastern Cape Provinces, South Africa, 2006–20010*

Predictor	Unadjusted analysis			Multivariate analysis	
	HR (95% CI)	p value		HR (95% CI)	p value
HIV positive	1.37 (1.09–1.72)	**0.005**		1.35 (1.06–1.72)	**0.02**
Previous MDR TB treatment	0.96 (0.76–1.23)	0.76		1.04 (0.77–1.42)	0.78
Smear positive	1.34 (1.07–1.68)	**0.01**		1.25 (0.97–1.62)	0.09
Age	0.99 (0.98–1.00)	0.25		0.100 (0.100–1.01)	0.69
Weight <50 kg	1.66 (1.33–2.08)	**<0.0001**		1.56 (1.23–1.98)	**0.0003**
KwaZulu Province	1.00 (0.78–1.28)	0.99		0.92 (0.67–1.26)	0.60
Male sex	0.92 (0.74–1.14)	0.43		0.99 *0.77–1.27)	0.94
Cavitary disease by chest radiograph	0.99 (0.78–1.26)	0.94		0.99 (0.76–1.30)	0.96

*p values in bold are statistically significant. HR, hazard ratio; MDR TB, multidrug-resistant tuberculosis.

## Discussion

For this large cohort of patients with XDR TB, we confirm previously reported poor outcomes in patients with or without HIV co-infection (*1**,**3**,**13*). Overall, 28.8% of patients were alive after 2 years of treatment, and 10.3% patients had a favorable outcome of cure or completion. This cohort survived a long duration of inadequate TB treatment before XDR TB treatment initiation, which is likely reflected in these results. Few patients with >6 months of previous MDR TB treatment were able to achieve a cure.

Patients in this study were in the first cohort of patients treated explicitly for XDR TB in South Africa. These patients were the first group to have access to additional second-line drugs, such as capreomycin and para-amino salicylic acid, as well as moxifloxacin in later instances. However, they were also survivors of previous failed or failing regimens for MDR TB treatment: patients were initiating XDR TB treatment a median of 4 months (IQR 0–8 months) after MDR TB treatment and an additional 8 months (IQR 5–12 months) after treatment for drug-susceptible TB (Table 1). As awareness has increased, patients have initiated appropriate TB treatment more rapidly. Furthermore, additional treatment options, such as linezolid, clofazimine, and bedaquiline and delamanid, have become available (*14**–**16*).

We found that patients with early culture conversion, within either 4 months or 8 months of treatment initiation, were not more likely to have a favorable outcome than patients with culture conversion after 8 months. Although overall treatment outcomes were poor, patients with culture conversion well into treatment (after 8–12 months) could still achieve cure. Conversely, patients without sputum culture conversion or with culture reversion could still survive 2 years of treatment and be counted as treatment successes. Although sputum culture conversion can be an appropriate surrogate endpoint for patients with drug-sensitive TB (*17*) and HIV-negative patients with MDR TB (*18*), it was not predictive in this study population.

When we assessed risk factors for favorable and unfavorable treatment outcomes, we found that HIV-positive patients receiving ARVs at start of treatment had similar treatment outcomes as patients not receiving ARVs. We take this finding as evidence that in this cohort of HIV-positive patients, ARVs were given too late in the disease course of a patient and that these patients received an XDR TB treatment regimen that was insufficient, such that poor outcomes were seen regardless. Multivariate analysis showed that HIV-positive patients were more likely to have an unfavorable outcome, but that HIV status was not a predictor of a favorable treatment outcome. We speculate that this discrepancy might be caused by difficulty in achieving a favorable outcome for XDR TB in any patient, especially the malnourished population of patients with chronic TB included in this cohort.

It likely that given the chronicity of disease and paucity of TB treatment options available in South Africa for the study cohort at the time of the study, patients were often unable to achieve cure by the time they were able to initiate appropriate treatment. This situation was true for patients who initiated treatment in October 2006, when XDR TB was first treated, as it was for patients who initiated treatment more than a year later, in January 2008.

Anecdotally, medication adherence during this study was suboptimal. Hospitalized patients were not always observed while taking medications; medications were often dispensed in cups for patients to take with meals. The consistency of DOT in long-term hospitalized patients has not been reported, but hospitalization should not be equated with DOT. Although policy prescribes DOT for all discharged patients, this policy is not always practical and feasible. Treatment incentive and enablers might improve treatment adherence (*19*) but were not available. Drugs for treatment of drug-resistant infections can cause severe side effects. In the absence of treatment support, patients might be more noncompliant than treatment records show. Imperfect DOT and limited support offered to patients might result in treatment interruptions, which could help further explain poor treatment outcomes.

Although access to HIV care improved during this time (*20*), only 1 new, possibly effective, drug, moxifloxacin, was made available during the study. Our finding of similarly poor results in HIV-negative and HIV-positive patients with or without HIV treatment underscores the WHO recommendation that >4 effective drugs be used in TB treatment. Furthermore, continued integration of vertical and horizontal health systems incorporating care for multiple disease processes is essential (*21*).

As in our study, previous research in resource-limited settings showed higher mortality rates in HIV-positive patients with smear-negative disease (*22**,**23*). A US study of HIV-positive patients with TB reported lower mortality rates in smear-negative case-patients, which the authors attributed to early-stage disease, given normal chest radiographic results for these patients (*24*). In that cohort, patients had a long history of previous treatment for drug-sensitive TB and MDR TB, and no difference was found among smear-positive and smear-negative patients.

The strength of our study was the large number of patients treated for XDR TB, many of whom were also HIV positive. Furthermore, repeated follow-up site visits while patients continued to receive treatment enabled us to reduce the problem of missing data, which can adversely affect retrospective and prospective series in resource-limited settings. Our cohort also provides context to initial reports in 2006 of XDR TB as a near universally fatal disease in Tugela Ferry, KwaZulu-Natal Province, where 98% of patients died in a median of 16 days (*2*).

Our study included only patients who survived to initiate treatment at the provincial hospital but showed that cure is still possible. In KwaZulu-Natal Province, an estimated 50%–70% of patients with MDR TB did not initiate treatment (*13**,**25*), but our previous study in Eastern Cape Province showed that 23.7% of patients did not survive to start treatment (*5*). By studying consecutive patients initiating treatment in 2 provinces, we could demonstrate some favorable outcomes. Our finding of less previous TB treatment in HIV-positive patients not receiving ARVs probably reflects this finding because many patients probably died before they were able to initiate appropriate treatment for either disease.

A further limitation of our study was that we were only able to assess HIV status and use of ARVs at the time of XDR TB treatment initiation and as dichotomous variables. These limitations represent a range of immunologic compromise and risk for death during treatment for XDR TB. More recent guidelines from 2013 state that all HIV-positive patients with any TB diagnosis should be given ARVs, which might have improved survival in our cohort (*26*). We found that HIV-positive patients receiving ARVs had better survival than patients not receiving ARVs, but HIV-positive patients were not more likely to achieve cure. The 2 HIV-positive patients not receiving ARVs who had a favorable treatment outcome initiated treatment when they had CD4+ counts >400 cells/mm^3^. Gandhi et al. reported a step-wise increase in mortality rates for HIV-positive patients with at least MDR TB and CD4 counts <50 cells/mm^3^, 50–200 cells/mm^3^, and >200 cells/mm^3^ (*13*).

Since 2008, when the last patients included in this research initiated treatment, many lessons have been learned in the management of XDR TB, resulting in programmatic and treatment changes. Since 2011, treatment for XDR TB has been decentralized in South Africa, and as of June 2015, a total of 400 sites were treating patients who had drug-resistant TB (*27*). Treatment regimens have been fortified by the addition of bedaquiline, delamanid, linezolid, clofazamine, and levofloxacin (*28*). Therefore, patients with XDR TB might be able to receive 3–5 effective drugs. Patients co-infected with drug-resistant TB and HIV are immediately given treatment for TB and HIV infection (*29*).

Our results demonstrate the need for rigorous follow-up of patients receiving treatment for TB. Just as MDR TB is caused by systemic failures in treatment for drug-susceptible TB, XDR TB develops when MDR TB is inadequately treated. Without >4 effective drugs in a TB treatment regimen and consistent adherence to medications, treatment cure is rare. These results, as well as those from Tugela Ferry in 2006 (*2*), illustrate worst-case scenarios in health systems. When TB or HIV infection are inadequately treated, diseases might spread rapidly and have lethal results.
