# Correction: Vol. 22, No. 9

**DOI:** 10.3201/eid2211.C32211

**Published:** 2016-11

**Keywords:** errata, erratum, correction

A second affiliation for author Martie L. van der Walt was omitted in Treatment Outcomes for Patients with Extensively Drug-Resistant Tuberculosis, KwaZulu-Natal and Eastern Cape Provinces, South Africa (C.L. Kvasnovsky et al.). The article has been corrected online (http://wwwnc.cdc.gov/eid/article/22/9/16-0084_article).

